# Effects of Pharmacist-Led Interventions Regarding Adult Patients with Type 2 Diabetes Mellitus in Mexico: A Systematic Review

**DOI:** 10.3390/pharmacy12050148

**Published:** 2024-09-27

**Authors:** Dulce D. Blanco-Vega, Alfonso Reyes-López, Jessica L. Vargas-Neri, Frida I. Osnaya-Valencia

**Affiliations:** 1 Department of Epidemiological Research, Hospital Infantil de México Federico Gómez, Mexico City 06720, Mexico; 313159196@quimica.unam.mx; 2 Faculty of Chemistry, Universidad Nacional Autónoma de México, Mexico City 04510, Mexico; 3 Center for Economic and Social Studies in Health, Hospital Infantil de México Federico Gómez, Mexico City 06720, Mexico; alreypez@hotmail.com

**Keywords:** pharmacist-led intervention, therapy management, type 2 diabetes, glycemic control, Mexico

## Abstract

In Mexico, type 2 diabetes mellitus (T2DM) is a serious public health concern. As experts in drug therapy, pharmacists are essential additions to multidisciplinary diabetes patient care teams. There have been no systematic reviews or meta-analyses performed on pharmacist-led interventions (PIs) in Mexico; therefore, the impact of PIs on patients remains poorly explored. An electronic search of the PubMed, SciELO and BVS databases and certain institutional repositories was conducted in English and Spanish through 24 August 2021 with a subsequent update through June 2024. A total of 1302 potentially relevant studies were identified in the initial search, of which nine met the eligibility criteria and were included in this systematic review. The results suggest that PIs, such as pharmacotherapeutic follow-up and patient education, may have positive effects on outcomes in Mexican patients with T2DM. PIs led to significant reductions in glycosylated hemoglobin, fasting blood glucose, triglycerides, total cholesterol, LDL cholesterol and arterial blood pressure levels, general reductions in body mass index and negative outcomes associated with medication, as well as significant improvements in therapeutic adherence and patient knowledge in the intervention group during follow-up periods of 3–12 months. Further well-designed research, including controlled studies with adequate sample sizes and standardized tools, is essential to fully understand the effects of PIs regarding patients with T2DM in Mexico.

## 1. Introduction

In Mexico, type 2 diabetes mellitus (T2DM) is a serious public health concern with significant morbidity and a profound impact on the functional capacity, health expenditure amount and quality of life of patients. In 2021, the International Diabetes Federation listed Mexico as the country with the world’s seventh highest number of adults with diabetes, with an estimated 14.1 million cases, the country with the sixth highest number of people with undiagnosed diabetes and the country with the eighth highest health expenditure worldwide, with approximately 19.9 billion USD in total diabetes-related spending [1].

T2DM has traditionally been progressively treated via establishing control goals and suitable modifications for the progression of chronic degenerative disease. Effective management involves both pharmacological and nonpharmacological treatments to regulate glucose levels and reduce the risk of microvascular and macrovascular complications [2]. As experts in drug therapy, pharmacists are essential additions to multidisciplinary diabetes patient care teams, given their extensive knowledge and ability to identify medication errors, adverse drug reactions and treatment specificities related to comorbidities, aging and polypharmacy. They also play a key role in providing patient education and bridging communication gaps between patients and other healthcare professionals.

Pharmacist-led interventions (PIs), including clinical pharmacy and pharmaceutical care activities, have been shown in multiple systematic reviews and meta-analyses to be useful for numerous conditions, such as diabetes, hypertension, hyperlipidemia and many other public health issues [3,4,5,6,7,8,9,10,11], with significant differences found in biochemical parameters, days of hospitalization, therapeutic adherence and a favorable cost-effectiveness ratio between patients who did and did not have access to PIs due to avoiding hospitalization costs, emergency department visits and savings in the utilization of medical care and pharmacy services. Nevertheless, publications on PIs usually gather information from high-income countries (HICs), and information is scarce from low- and middle-income countries (LMICs), such as Mexico, Brazil, Chile and Argentina [12]. In fact, the current literature does not include any data from Mexico, and there have been no systematic reviews or meta-analyses published on PIs in Mexico. This is mainly because, in Mexico, clinical pharmacy and pharmaceutical care remain relatively new and underdeveloped disciplines [13]. The role of the clinical pharmacist is seldom incorporated into the patient care team, resulting in a limited understanding of the impact of PIs on patient outcomes. Integrating clinical pharmacists into the care of patients with T2DM is crucial for its inclusion as a health professional in direct patient care.

The aim of this systematic review was to identify the effects of pharmacist-led interventions on biochemical parameters, therapeutic adherence, drug-related problems, negative outcomes associated with medication and level of knowledge of adult patients diagnosed with T2DM in Mexico. The results of this work support further investigation of the extremely limited clinical pharmacy and pharmaceutical care activities performed to date in Mexico.

## 2. Materials and Methods

### 2.1. Data Sources

An electronic search of published studies was conducted in English and Spanish through 24 August 2021, following the PRISMA guidelines [14] and using the PubMed, SciELO and BVS electronic databases, as well as the institutional repositories of theses from the Universidad Nacional Autónoma de México (UNAM) and the Benemérita Universidad Autónoma de Puebla (BUAP). Reference lists were also manually analyzed to find additional eligible studies that were not identified through the database searches. Additionally, a subsequent update of the search strategies was carried out until June 2024.

The databases were searched for the following key terms: “clinical pharmacy”, “pharmaceutical care”, “pharmacotherapeutic follow-up”, “pharmacist intervention”, “diabetes”, “Mexico”, and “Mexican patient”. These key terms were combined by the Boolean operators “AND” and “OR” where appropriate. Each search strategy considered and conducted in English and Spanish is detailed in Table S1 of the supplementary material.

### 2.2. Study Selection

Studies were considered for inclusion if they quantified the effects of PIs delivered in Mexico via any route and in any setting directly to adult patients with T2DM or to healthcare professionals caring for these patients who measured the effects of PIs on glycosylated hemoglobin (HbA1c) and fasting blood glucose (FBG) levels as primary endpoints and the following secondary endpoints: lipid profiles, systolic and diastolic blood pressure, weight loss, urine albumin/creatinine ratios, risks of coronary heart disease, risks of stroke, self-care activities such as diet and exercise, cost-effectiveness ratios (quality-adjusted life years (QALYs)), hospitalizations, morbidity, mortality, drug-related problems, negative outcomes associated with medication (NOMs), adverse drug reactions, health-related quality of life, therapeutic adherence, level of knowledge and patient satisfaction. All studies for which the full text could not be accessed, secondary studies such as systematic reviews and meta-analyses, descriptive studies, studies where nonclinical pharmaceutical activities were performed (storage, dispensing, etc.) or those in which activities were not performed by a pharmacist, were excluded from the systematic review.

Two reviewers (D.D.B.-V., F.I.O.-V.) independently selected the articles, first by title and abstract with a Cohen’s kappa of 0.68 (substantial agreement among reviewers [15]) and then by full text with a Cohen’s kappa of 0.92 (almost perfect agreement among reviewers [15]). Discrepancies were resolved by a third reviewer (J.L.V.-N.). The process of importing all the articles returned by the search strategies, eliminating duplicates, selecting studies and extracting the data were conducted using Covidence^®^ web-based systematic review software.

### 2.3. Data Extraction and Assessment of Bias Risk

The following data were extracted in the standard Covidence^®^ format using Microsoft^®^ Excel: authors and year of publication, type of study, patient characteristics, PIs, follow-up period, outcomes and key findings or conclusions. The ROBINS-I (Risk of Bias in Nonrandomized Studies of Interventions) Cochrane tool for nonrandomized studies [16] was used to determine whether the included studies had low, moderate, severe or critical risks of bias based on its detailed guidelines [17]. For the randomized clinical trial, the Revised Cochrane Risk-of-Bias Tool for Randomized Trials (RoB 2) [18] was used to evaluate the risk of bias, classifying the study as having low risk of bias, some concerns or high risk of bias. In parallel, the standardized DEPICT (Descriptive Elements of Pharmacist Interventions Characterization Tool) system [19] was used to describe and identify the elements that constituted each PI.

## 3. Results

### 3.1. Selection of Studies

**Figure 1:**
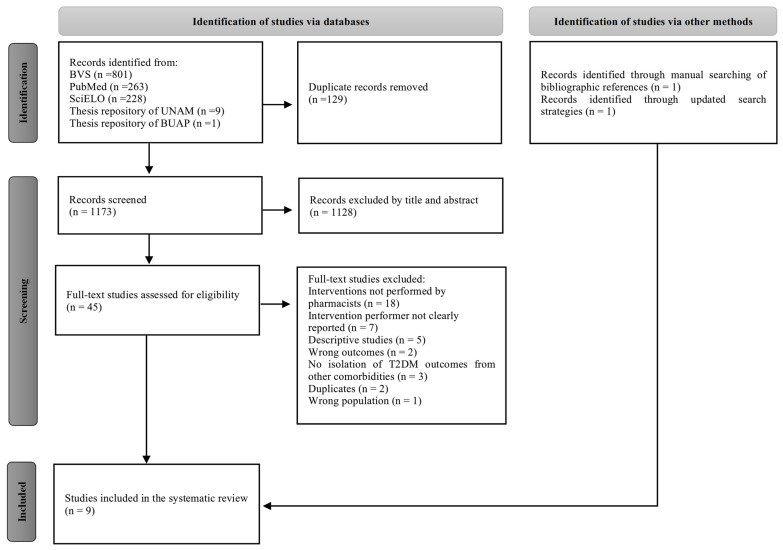
PRISMA flow diagram of the study selection process.

A total of 1302 potentially relevant studies were identified in the initial search (Figure 1). After removal of 129 duplicates, 1128 studies were excluded by title and abstract and 38 were subsequently excluded by full text, mainly because the pharmacist was not involved in the interventions or because the practitioner who delivered the intervention was not specified. Finally, seven studies were identified through the databases and two additional studies were subsequently manually identified, resulting in a total of nine studies included in this systematic review.

### 3.2. Overview of the Included Studies

The main characteristics of the included studies are presented in Table 1. Only one randomized clinical study with low risk of bias was found [20]. The remaining studies were quasiexperimental, although only three of them were controlled for a moderate-to-severe risk of bias [21,22,23], and the five remaining uncontrolled studies presented critical risks of bias. The PIs focused on pharmacotherapeutic follow-up through the Dader Method in combination with patient education (67%) [21,22,24,25,26,27]; in three studies [20,23,28], patient education was exclusively performed (33%), with a follow-up period from 3 to 12 months with 3–6 months being the most frequent time of follow-up. The measurements of primary outcomes included HbA1c and FBG levels and those of secondary outcomes included triglyceride levels, total cholesterol (TC) levels, LDL cholesterol levels, HDL cholesterol levels, arterial blood pressure, body mass index (BMI), adherence to pharmacological treatment, NOM and level of knowledge. In the controlled studies, the control group typically received standard care. However, in one study [23], in conjunction with standard care, patients also received a brochure with basic information on the use of T2DM treatments and antihypertensive drugs.

### 3.3. Characteristics of Included Study Populations

As detailed in Table 1, the study populations were between 24 and 83 years of age, with the most frequently assessed group being individuals between 50 and 60 years of age, mostly female, and having comorbidities of arterial hypertension, overweight or obesity. Only one study [21] reported a population with no comorbidities, while two studies [27,28] did not specify whether their populations had comorbidities. Overall, the study populations typically had a low level of education (i.e., education up to or including elementary school). However, one study [23] reported that its population primarily had a medium level of education (i.e., education ranging from middle school to high school).

### 3.4. Characteristics of Pharmacist-Led Interventions

In the pharmacotherapeutic follow-up, pharmacists recommended modifications to the pharmacological strategy, including adjustments to dose, frequency and duration of treatment, as well as the addition or discontinuation of medications. In terms of patient education, topics covered included T2DM and its complications, psychological aspects, both pharmacological and nonpharmacological treatment strategies, dietary habits, physical activity, proper medication use and storage, adherence to treatment, actions to take in case of missed doses and adverse reactions. Additionally, the authors reported providing support materials such as educational slides validated by experts, brochures, posters, appointment and identification cards, medication schedules, pill organizers, alarms, and prescriptions converted into pictograms.

The interventions were carried out by pharmacists. In one study [28], the interventions were part of a multidisciplinary team, while in another study [21], pharmacy students and pharmacy assistants, under the supervision of a pharmacist, conducted the interventions. The recipients of the interventions were primarily patients, who were contacted face-to-face, either individually or in groups. Only in one study [24], the intervention also involved a physician, who was contacted both face-to-face and in writing. Most interventions occurred during outpatient care at family medicine units through scheduled appointments or following the patients’ medical consultations. Additionally, in three studies [21,23,25], interventions were conducted at patients’ homes, and in one study [25], they were also carried out in a mobile unit specifically designed for interventions (Table 2). 

### 3.5. Study Outcomes

#### 3.5.1. Biochemical Parameters

Following PI performance in the intervention group, significant decreases (*p* < 0.05) in HbA1c [20,22,24], FBG [20,21,26,27], triglycerides [20,22,27], systolic and diastolic blood pressure [20], TC [20,21,27] and LDL cholesterol [21] levels were reported, in addition to a statistically significant increase in HDL cholesterol levels [21] and general reductions in BMI [22] (Table 1).

#### 3.5.2. Therapeutic Adherence, NOM and Level of Knowledge

Five of the nine studies evaluated adherence to pharmacological treatment [20,21,23,25,26]. Among these, four studies [20,21,25,26] reported increases in adherence ranging from 5% to 43.5% after intervention, as measured via the Morisky–Green–Levine test (MGL), with three of these studies showing significant increases [20,21,26]. In contrast, one study assessed adherence using patient pill counts and no significant differences were reported [23] (Table 1).

Only two of the nine studies evaluated NOM after intervention [21,26], and only one study [26] reported significant decreases in nonquantitative infectivity (NQI) and quantitative ineffectiveness (QIF). Two of the nine studies evaluated changes in knowledge levels after the interventions [22,28], finding significant increases using the Diabetes Knowledge Questionnaire 24 [28] and other questionnaires on habits and lifestyle and on knowledge of the disease and treatment [22] (Table 1).

## 4. Discussion

### 4.1. Main Findings

Our systematic review results revealed a positive trend for PIs in Mexico, including pharmacotherapeutic follow-up and patient education, with significant decreases in HbA1c, FBG, triglycerides, TC, LDL cholesterol and arterial blood pressure levels, as well as a statistically significant increase in HDL cholesterol levels, therapeutic adherence and the level of knowledge among patients with T2DM. However, although the results are consistent with those reported in the literature from systematic reviews and meta-analyses [29,30,31,32,33], primarily based on data from HICs, this review’s findings are limited by the small number of studies, most of which have weak experimental designs and moderate to critical risks of bias.

Meta-analysis of the included studies was explored, and although an effect suggesting a potential benefit of PIs was observed, it was decided not to present these results, as only three controlled studies were feasible to be included, representing too small a sample size to draw a solid conclusion.

### 4.2. PIs in Mexico

According to this systematic review, the PIs in Mexico were focused on pharmacotherapeutic follow-up in combination with patient education or patient education alone and were delivered mainly face-to-face to patients in outpatient care in family medicine units or in patients’ homes. This is particularly relevant due to the more specific effect of delivering the intervention directly to the patient rather than solely to the healthcare professional. Additionally, the study population had a generally low level of education and given the established relationship between low education levels and poor adherence to therapy [34,35], this emphasizes the importance of patient education as a key component of PIs for achieving therapeutic goals.

In other countries, pharmacists have the autonomy to adjust insulin doses and therefore to perform this as a PI [11], which is in great contrast to Mexico, where the pharmacist does not have the autonomy to prescribe. Furthermore, PIs are developed in a range of settings abroad, including community pharmacies, primary care clinics, health centers and hospitals [29,30,31,32,33]. In Mexico, however, there are few places where pharmacists have been able to develop clinical pharmacy and pharmaceutical care interventions. Moreover, Mexican society still does not fully recognize the role of pharmacists in patient care activities.

The small number of studies included in the systematic review may be attributed to several factors. The education of clinical pharmacy and pharmaceutical care in Mexico is limited. Not all Mexican universities adhere to the international guidelines set by the International Pharmaceutical Federation for pharmacist education. Moreover, the teaching of clinical pharmacy and pharmaceutical care is not included in the curricula of several universities and specializations that enhance pharmacists’ performance in patient care are scarce [36,37]. Additionally, a historical lack of regulation and multiple health code reforms has led to the pharmacy practice without an academic degree in Mexico, as well as a loss of workspace in pharmacies due to pharmacy assistants, pharmacy interns or counter employees with scarce general knowledge of pharmaceutical care and pharmacy [38,39]. Furthermore, a limited regulatory framework with major areas for improvement in the concepts and consolidation of clinical pharmacy and pharmaceutical care have contributed to creating an extremely hostile environment for the implementation of PIs and for the integration of clinical pharmacists in multidisciplinary patient care teams in Mexico. It is imperative to strengthen collaboration between the academic sector and the Mexican government to standardize concepts and ensure the professional education of clinical pharmacists. This will facilitate the effective implementation of PIs and improve patient care.

Despite the limited number of studies included in the systematic review and their methodological weaknesses, it was observed that PIs tend to have positive effects on various clinical outcomes in patients with T2DM. This is especially relevant in Mexico, which is among the top ten countries with the highest number of diabetes cases and the highest diabetes-related expenditures. The increasing prevalence of diabetes and related mortality presents unprecedented challenges for the Mexican health system, particularly in managing chronic diseases like T2DM, which requires continuous healthcare.

Well-designed research, including controlled studies with adequate sample sizes and standardized tools, is essential to fully understand the impact of PIs not only in Mexico but across Latin America, where pharmacists appear to continue facing challenges in demonstrating their significant positive impact on public health. The evaluation of well-designed interventional prospective studies on the impact of PIs on days of hospitalization, morbidity, mortality and cost-effectiveness must also be considered. Finally, more studies are needed to assess the long-term effects of PIs in patients with T2DM. There is still a lack of evaluation regarding whether these interventions prevent diabetes-related microvascular and macrovascular complications, such as kidney failure and diabetic foot. Such studies are undoubtedly necessary to demonstrate that PIs could contribute to making healthcare systems more sustainable. Nevertheless, the development of these studies will first rely on strengthening PIs in Mexico. 

### 4.3. Limitations

The major limitation of this systematic review is the limited number of studies that could be included, as well as the low quality of evidence and small sample sizes in most of them. This limitation means that we can only describe specific trends regarding the impact of PIs on patients with T2DM in Mexico. Due to the nature of the data, we could not identify the specific effects of PIs on other important outcomes such as quality of life, adverse events, days of hospitalization, mortality and costs. Additionally, the focus of this systematic review solely on Mexico means that the findings cannot be extrapolated to other countries. However, this review aimed to identify the effects of PIs in patients with T2DM in Mexico so that future reviews can compare these interventions in Mexico with those in other countries, particularly in Latin America, and enhance their implementation among multidisciplinary teams caring for patients.

Another limitation of this review is that it was not registered in the international database PROSPERO; however, it was conducted according to PRISMA guidelines and with the support of Covidence software to minimize any bias in the development of the systematic review.

Finally, regarding the descriptions of PIs, the authors generally provided the basic elements for their characterization; however, as shown in Table 2, there are still elements that are not reported, which represents a limitation in the analysis and reproducibility of these PIs. The use of standardized and validated tools, such as the DEPICT tool, to guide the description of the components of PIs is crucial for their reporting, analysis, comparison and potential implementation.

## 5. Conclusions

This systematic review suggests that pharmacist-led interventions, such as pharmacotherapeutic follow-up and patient education, may have positive effects on outcomes in Mexican patients with T2DM, as seen in other countries, by significantly reducing glycosylated hemoglobin, fasting blood glucose, triglycerides, total cholesterol, LDL cholesterol and arterial blood pressure levels, generally reducing body mass index and negative outcomes associated with medication, as well as significantly improving therapeutic adherence and patient knowledge in the intervention group during follow-up periods of 3–12 months. Given the limitations of the current dataset, further research is needed to confirm these findings and enhance their applicability.

## Figures and Tables

**Table 1:** Overall characteristics of the included studies.

Reference	Settings	Population	Study Design	Pharmacist Intervention	Follow-Up Period (Months)	Patient Outcomes				Risk of Bias
						Outcome	C	I		
Contreras-Vergara et al., 2022 [20]	Ambulatory care	Type 2 diabetes; age (years): 18–60 (mean C: 55.8 ± 3.6, I: 56.6 ± 2.3); C: 60.5% female, I: 61.5% female; C: 67.4% low level of education (23.3% medium level of education, 9.3% high level of education), I: 67.4% low level of education (26.1% medium level of education, 6.5% high level of education); comorbidities AH	Randomized clinical trial (standard care *n* = 43)	Patient education (*n* = 46)	6	HbA1c	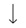	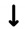		Low risk of bias
						FBG	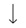	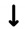		
						Triglycerides	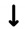	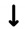		
						TC	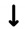	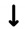		
						Systolic blood pressure	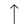	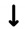		
						Diastolic blood pressure	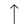	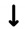		
						Therapeutic adherence (MGL)	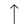	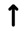		
Sosa et al., 2014 [21]	Patients’ homes	Type 2 diabetes; age (years): 24–83 (mean 52); 94% female; level of education not reported; no comorbidities	Quasiexperimental, controlled (standard care *n* = 36)	Pharmacotherapeutic follow-up (Dader Method) and patient education (*n* = 41)	12S	HbA1c	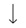	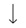		Moderate risk
						FBG	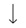	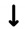		
						Triglycerides	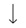	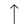		
						TC	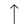	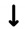		
						LDL cholesterol	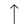	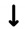		
						HDL cholesterol	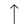	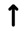		
						Therapeutic adherence (MGL)	NM	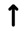		Critical risk
						NOM	NM	N *	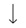	
								NQI	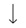	
								QIF	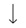	
								QIS	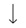	
González-Herrera, 2012 [22]	Ambulatory care	Type 2 diabetes; age (years): 25–65; 90% female; 75% low level of education; comorbidities AH, HL, CVD, KD	Quasiexperimental, controlled (standard care *n* = 10)	Pharmacotherapeutic follow-up (Dader Method) and patient education (*n* = 12)	6	HbA1c	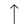	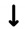		Moderate risk
						FBG	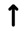	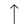		
						Triglycerides	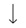	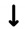		
						TC	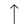	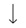		
						Systolic blood pressure	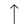	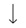		
						Diastolic blood pressure	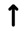	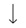		
						BMI	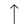	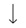		
						LOK (habits and lifestyle) **	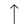	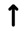		
						LOK (disease and treatment) **	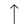	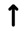		
Mino-León et al., 2014 [23]	Ambulatory care/ patients’ homes	Type 2 diabetes; mean age: 58 years; 56% medium level of education; comorbidities AH, HL, OB, OA, CM, KD.	Quasiexperimental, controlled (standard care plus a brochure on the use of medications *n* = 152)	Patient education (*n* = 137)	3	Glycemic control	Intervention group showed a 13% higher possibility of achieving glycemic control (≤130 mg/dL) compared to the control group.			Moderate risk
						Therapeutic adherence (pill count)	No statistically significant difference: OR = 1.27 (95% CI: 0.80–2.02)			
Reynoso-Zárate, 2015 [24]	Ambulatory care	Type 2 diabetes; age (years): 43–72 (mean 60); 60% female; level of education not reported; comorbidities OW, OB	Quasiexperimental, uncontrolled	Pharmacotherapeutic follow-up (Dader Method) and patient education (*n* = 15)	3–6	HbA1c	N/A	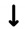		Critical risk
						FBG	N/A	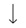		
Juárez-Cano, 2015 [25]	Ambulatory care/ patients’ homes	Type 2 diabetes; age (years): 40–80; 84% female; level of education not reported; comorbidities AH, OW, OB	Quasiexperimental, uncontrolled	Pharmacotherapeutic follow-up (Dader Method) and patient education (*n* = 19)	3	HbA1c	N/A	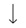		Critical risk
						FBG	N/A	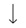		
						Triglycerides	N/A	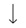		
						TC	N/A	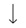		
						Therapeutic adherence (MGL)	N/A	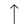		
Toledano et al., 2012 [26]	Ambulatory care	Type 2 diabetes; age (years): 37–87 (mean 61); 66% female; level of education not reported; comorbidities AH, OW, OB	Quasiexperimental, uncontrolled	Pharmacotherapeutic follow-up (Dader Method) and patient education (*n* = 71)	6	FBG	N/A	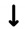		Critical risk
						Therapeutic adherence (MGL)	N/A	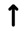		
						NOM	N/A	N *	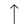	
								NQI	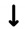	
								QIF	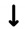	
								QIS	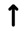	
Herrera-Huerta et al., 2012 [27]	Not reported	Type 2 diabetes; mean age: 58 years; level of education not reported; comorbidities not reported	Quasiexperimental, uncontrolled	Pharmacotherapeutic follow-up (Dader Method) and patient education (*n* = 145)	3–6	FBG	N/A	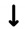		Critical risk
						Triglycerides	N/A	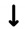		
						TC	N/A	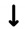		
López et al., 2006 [28]	Not reported	Type 2 diabetes; age (years): 39–72 (mean 53); 65% female; 65% low level of education; comorbidities not reported	Quasiexperimental, uncontrolled	Patient education (*n* = 17)	6	HbA1c	N/A	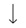		Critical risk
						LOK (DKW-24) **	N/A	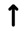		

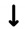
 Statistically significant decrease, 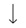 not statistically significant decrease. 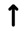 Statistically significant increase, 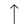 not statistically significant increase, C: control group, I: intervention group, NM: no measurement was performed, N/A: not applicable since there was no control group. * Types of negative outcomes associated with medication (NOM): N: necessity, NQI: nonquantitative ineffectiveness, QIF: quantitative ineffectiveness, QIS: quantitative insecurity. ** The knowledge questionnaires are composed of several sections; thus, the results shown correspond to an increase or decrease in all or most of the sections. Low level of education: education less than or equal to elementary school, medium level of education: education ranging from middle school to high school, high level of education: education greater than or equal to college. AH: arterial hypertension, BMI: body mass index, CM: cardiomyopathy, CVD: cerebrovascular disease, DKQ-24: Diabetes Knowledge Questionnaire 24, FBG: fasting blood glucose, HbA1c: glycosylated hemoglobin, HL: hyperlipidemia, KD: kidney disease, LOK: level of knowledge, MGL: Morisky–Green–Levine test, NOM: negative outcomes associated with medication, OA: osteoarthritis, OB: obesity, OW: overweight, TC: total cholesterol.

**Table 2:** Characterization of the descriptive elements of pharmacist interventions through the DEPICT tool.

Reference	Intervention Performer	Contact with Recipient	Setting	Clinical Data Sources	Variables Assessed	Interventions	Supplementary Material
Contreras-Vergara et al., 2022 [20]	Pharmacist	One-on-one and face-to-face (patient)	Ambulatory setting; scheduled appointments	Medication lists, adherence measuring tools and patient interviews	Medication adherence and patient nutrition or lifestyle	Patient education on T2DM characteristics and complications, nutrition, exercise, therapeutic adherence, and the usefulness of pharmacological treatments	Educational leaflets, wallet card listing prescription medications
Sosa et al., 2014 [21]	One pharmacist, one pharmacy technician and two pharmacy students	One-on-one and face-to-face contact with group (patient)	Patients’ homes; scheduled appointments	Drug prescription orders, medication lists, adherence measuring tools, laboratory tests and patient interviews	Drug selection, medication effectiveness, medication safety and medication adherence	Pharmacotherapeutic follow-up (Dader Method), modification of pharmacological strategy; patient education about the pathology, risk factors, pharmacological treatment, self-control, self-care and self-monitoring	Educational audiovisual material
González-Herrera, 2012 [22]	Pharmacist	One-on-one and face-to-face (patient)	Ambulatory setting; scheduled appointments	Drug prescription orders, medication lists, laboratory tests and patient interviews	Drug selection, medication effectiveness, medication safety and patient nutrition or lifestyle	Pharmacotherapeutic follow-up (Dader Method); patient education about pathology and its complications, importance of compliance with the dosage regimen; influence of medications on treatment, nutritional habits and physical activity	Educational leaflets, posters and patient appointment cards
Mino-León. 2014 [23]	Pharmacist	One-on-one and face-to-face (patient)	Patients’ homes, ambulatory setting; after medical appointment and scheduled appointments	Medication lists and adherence measuring tools	Medication adherence	Patient education on pharmacological and nutritional aspects, adverse reactions, importance of taking medications, actions to take in case of missing doses and medication counseling	Pictorial instructions
Reynoso-Zárate, 2015 [24]	Pharmacist	One-on-one and face-to-face (patient); One-on-one, face-to-face and written (physician)	Ambulatory setting; scheduled appointments	Drug prescription orders, medication lists, laboratory tests, patient interviews and medical records	Drug selection, medication effectiveness and medication safety	Pharmacotherapeutic follow-up (Dader Method), dosage and treatment modifications; patient education on pathology, pharmacotherapy, medication adherence, nutritional habits and physical activity	Educational leaflets
Juárez-Cano, 2015 [25]	Pharmacist	One-on-one and face-to-face (patient)	Mobile care unit and patients’ homes; scheduled appointments	Medication lists, adherence measuring tools, laboratory tests and patient interviews	Drug selection, medication effectiveness, medication safety, medication adherence and patient nutrition or lifestyle	Pharmacotherapeutic follow-up (Dader Method), addition, suspension, substitution or adjustment of medication doses; referral to a nephrologist and internist, patient education on pathology, correct administration of medications, storage and medication adherence, nutrition habits and physical activity	Written action plan and medication compliance device
Toledano et al., 2012 [26]	Pharmacist	One-on-one and face-to-face (patient)	Ambulatory setting; after medical appointment and scheduled appointments	Medication lists, adherence measuring tools, laboratory tests, patient interviews and medical records	Drug selection, medication effectiveness, medication safety and medication adherence	Pharmacotherapeutic follow-up (Dader Method), modification of doses, changes in the frequency and/or duration of treatment and addition of medication; patient education on pharmacological treatment, nonpharmacological measures and medication use	Educational materials, auxiliary labels, medication schedules, and medication compliance device
Herrera-Huerta et al., 2012 [27]	Pharmacist	Not reported	Not reported	Not reported	Drug selection, medication effectiveness and medication safety	Pharmacotherapeutic follow-up (Dader Method), patient education on disease management, proper use of medications and their importance; self-care, diets, exercise and risk factors	Educational leaflets and posters
López et al., 2006 [28]	Multidisciplinary team (physician, psychologist, nutritionist and a pharmacist)	One-on-one and face-to-face contact with group (patient)	Not reported	Not reported	Not reported	Patient education on psychological, nutritional, medical, pharmacological aspects and the main acute and chronic complications of T2DM	Not reported

## Data Availability

Not applicable.

## Supplementary Materials

The following supporting information can be downloaded at: https://www.mdpi.com/article/10.3390/pharmacy12050148/s1, Table S1: Full database search strategies.
