# Supplementary material for: Effects of Pharmacist-Led Interventions Regarding Adult Patients with Type 2 Diabetes Mellitus in Mexico: A Systematic Review
Source: Pharmacy (Basel). 2024 Sep 27;12(5):148. doi: 10.3390/pharmacy12050148 (PMC11511297; doi:10.3390/pharmacy12050148)
Supplement: Supplementary file 1 [file pharmacy-12-00148-s001.zip › pharmacy-3157296-supplementary.pdf]

pharmacy MDPI

Table S1. Full database search strategies.

PubMed
(("clinical pharmacy") OR (telemedicine) OR ("pharmaceutical care") OR ("pharmacy services") OR ("pharmaceutical services") OR ("Pharmacotherapeutic Follow-up") OR ("medication therapy management"[Mesh Terms]) OR ("Drug-Related Side Effects and Adverse Reactions" [Mesh Terms]) OR ("Counseling" [Mesh Terms]) OR ("Medication Reconciliation" [Mesh Terms]) OR ("Patient Education as Topic" [Mesh Terms]) OR ("drug therapy services") OR ("drug therapy adjustment") OR (Pharmacist) OR ("Pharmacist intervention")) AND ((diabetes) OR ("diabetes mellitus, type 2"[MeSH Terms]) OR ("blood glucose") OR (glycemia) OR ("Glycemic Treatment") OR ("Glycemic control") OR ("Glycated Hemoglobin A"[MeSH Terms]) OR ("glycosylated hemoglobin")) AND ((Mexico) OR ("Mexican patient"))
**SciELO (Spanish search)**
(("atención farmacéutica") OR ("programa de atención farmacéutica") OR ("farmacia clínica") OR ("servicios farmacéuticos") OR ("farmacia comunitaria") OR ("farmacia hospitalaria") OR ("intervenciones farmacéuticas") OR ("intervención farmacéutica") OR ("actuación farmacéutica") OR (Farmacéutico) OR (telemedicina) OR ("intervención educativa") OR ("Seguimiento farmacoterapéutico") OR ("Seguimiento farmacéutico") OR ("metodología Dáder") OR ("método Dáder") OR ("educación sanitaria") OR ("educación al paciente") OR ("adherencia a la medicación") OR ("adherencia al tratamiento") OR ("adherencia del paciente") OR ("adherencia terapéutica") OR ("resultados negativos asociados a la medicación") OR ("problemas relacionados con medicamentos") OR ("reacciones adversas") OR ("Nivel de conocimientos") OR ("grado de conocimientos") OR ("estilo de vida")) AND (México)
**BVS (Spanish search)**
(("diabetes mellitus tipo 2") OR ("diabetes tipo 2") OR (diabetes$) OR ("control glicémico") OR ("atención de la diabetes") OR ("control metabólico") OR (DM2) OR ("pacientes diabéticos") OR ("poblaciones diabéticas") OR (glucemia) OR ("hemoglobina glicosilada") OR ("hemoglobina glucosilada")) AND (("atención farmacéutica") OR ("programa de atención farmacéutica") OR ("farmacia clínica") OR ("servicios farmacéuticos") OR ("farmacia comunitaria") OR ("farmacia hospitalaria") OR ("intervenciones farmacéuticas") OR ("intervención educativa") OR ("intervención farmacéutica") OR ("actuación farmacéutica") OR (Farmacéutico) OR (telemedicina) OR ("Seguimiento farmacoterapéutico") OR ("Seguimiento farmacéutico") OR ("metodología Dáder") OR ("método Dáder") OR ("educación sanitaria") OR ("educación al paciente") OR ("adherencia a la medicación") OR ("adherencia al tratamiento") OR ("adherencia del paciente") OR ("adherencia terapéutica") OR ("resultados negativos asociados a la medicación") OR ("problemas relacionados con medicamentos") OR ("reacciones adversas") OR (Hospitalización) OR (mortalidad) OR ("Nivel de conocimientos") OR ("grado de conocimientos") OR ("estilo de vida")) AND (México)
**Thesis repository of the Universidad Nacional Autónoma de México (UNAM) (Spanish search)**
Individual search: "intervención farmacéutica", "atención farmacéutica", "seguimiento farmacoterapéutico"
**Thesis repository of the Benemérita Universidad Autónoma de Puebla (BUAP) (Spanish search)**
Individual search: "Seguimiento farmacoterapéutico"
